# Ethyl 2-[4-(2-chloro­benzo­yl)-2,6-di­methyl­phen­oxy]ethano­ate

**DOI:** 10.1107/S1600536812030693

**Published:** 2012-07-14

**Authors:** T. Prashanth, V. Lakshmi Ranganatha, M. K. Usha, Shaukath Ara Khanum, D. Revannasiddaiah, Sumati Anthal, Rajni Kant, Vivek K. Gupta

**Affiliations:** a Department of Chemistry, Yuvaraja’s College, University of Mysore, Mysore 570 005, India; b Department of Studies in Physics, University of Mysore, Mysore 570 006, India; c Post-Graduate Department of Physics and Electronics, University of Jammu, Jammu Tawi 180 006, India

## Abstract

The asymmetric unit of the title compound, C_19_H_19_ClO_4_, contains two independent mol­ecules. The dihedral angles between the benzene rings are 63.41 (8) and 61.41 (9)°. Adjacent mol­ecules of different types are inter­connected in pairs through π–π inter­actions between their central benzene rings [centroid–centroid separation = 3.801 (2) Å, inter­planar spacing = 3.605 (2) Å, centroid shift = 1.204 (2) Å]. Finally, C—H⋯O hydrogen bonds link these dimers into bilayers parallel to (100).

## Related literature

For general background to phen­oxy­ethanoic acid, see: Dahiya & Kaur (2007 ▶); Esbenshade *et al.* (1990 ▶). For biological activity, see: Prabhakar *et al.* (2006 ▶); Sudha *et al.* (2003 ▶); Ma *et al.* (2011 ▶); Khanum *et al.* (2010 ▶). For bond-length data, see: Allen *et al.* (1987 ▶).
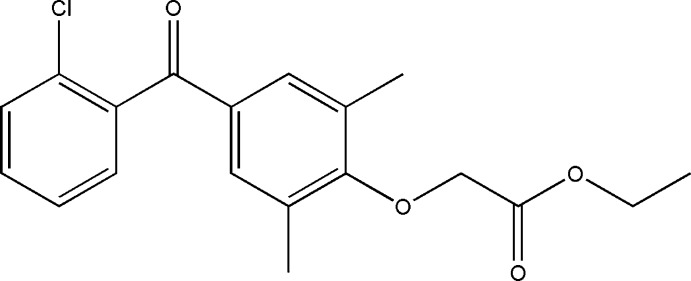


## Experimental

### 

#### Crystal data

C_19_H_19_ClO_4_
*M* *_r_* = 346.79
Monoclinic, 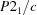

*a* = 16.4082 (8) Å
*b* = 14.7290 (6) Å
*c* = 15.4470 (8) Å
β = 107.268 (5)°
*V* = 3564.9 (3) Å^3^
*Z* = 8
Mo *K*α radiation
μ = 0.23 mm^−1^
*T* = 293 K
0.30 × 0.20 × 0.20 mm

#### Data collection

Oxford Diffraction Xcalibur Sapphire3 diffractometer
Absorption correction: multi-scan (*CrysAlis PRO*; Oxford Diffraction, 2010 ▶) *T*_min_ = 0.912, *T*_max_ = 1.000
17025 measured reflections
6997 independent reflections
3576 reflections with *I* > 2σ(*I*)
*R*_int_ = 0.039

#### Refinement

*R*[*F* ^2^ > 2σ(*F* ^2^)] = 0.064
*wR*(*F* ^2^) = 0.191
*S* = 1.02
6997 reflections
439 parameters
H-atom parameters constrained
Δρ_max_ = 0.46 e Å^−3^
Δρ_min_ = −0.36 e Å^−3^

### 

Data collection: *CrysAlis PRO* (Oxford Diffraction, 2010 ▶); cell refinement: *CrysAlis PRO*; data reduction: *CrysAlis PRO*; program(s) used to solve structure: *SHELXS97* (Sheldrick, 2008 ▶); program(s) used to refine structure: *SHELXL97* (Sheldrick, 2008 ▶); molecular graphics: *ORTEP-3* (Farrugia, 1997 ▶); software used to prepare material for publication: *PLATON* (Spek, 2009 ▶).

## Supplementary Material

Crystal structure: contains datablock(s) I, global. DOI: 10.1107/S1600536812030693/bg2469sup1.cif

Structure factors: contains datablock(s) I. DOI: 10.1107/S1600536812030693/bg2469Isup2.hkl

Supplementary material file. DOI: 10.1107/S1600536812030693/bg2469Isup3.cml

Additional supplementary materials:  crystallographic information; 3D view; checkCIF report

## Figures and Tables

**Table 1:** Hydrogen-bond geometry (Å, °)

*D*—H⋯*A*	*D*—H	H⋯*A*	*D*⋯*A*	*D*—H⋯*A*
C11*A*—H11*A*⋯O18*B* ^i^	0.93	2.49	3.346 (6)	153
C11*B*—H11*B*⋯O18*A* ^i^	0.93	2.47	3.351 (5)	159
C14*B*—H14*B*⋯O9*A*	0.93	2.59	3.482 (4)	161
C20*A*—H20*A*⋯O9*B* ^ii^	0.97	2.57	3.420 (5)	147

Symmetry codes: (i) 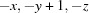; (ii) 
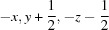
.

**Fig. 1.**
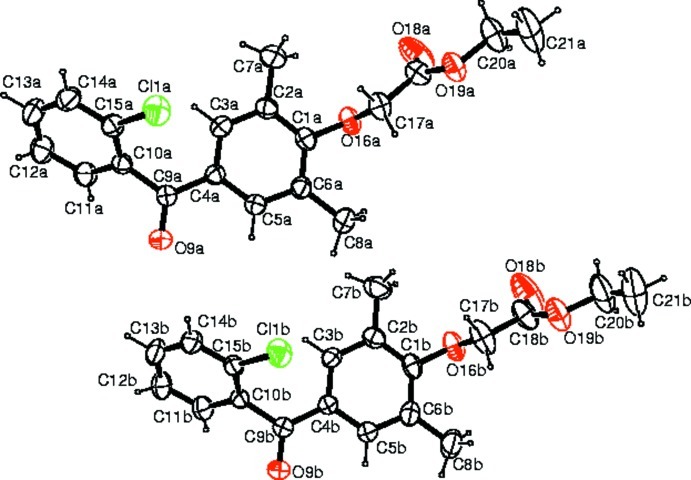
ORTEP view of the molecule with the atom-labeling scheme. The thermal ellipsoids are drawn at the 40% probability level. H atoms are shown as small spheres of arbitrary radii.

## supplementary crystallographic information

### Comment

Analogues of phenoxy ethanoic acid are considered to be very important compounds in the field of medicinal chemistry, and the compounds were found to have good antifungal activity against pathogenic fungi and posses moderate activity against gram negative bacteria in comparison to standard ciprofloxacin (Dahiya & Kaur 2007). Resent studies shows that changes in the chemical and stereoisomeric structures of phenoxy ethanoic acid alter peroxisome proliferation (Esbenshade *et al.*, 1990). The anti-inflammatory activity results revealed a significant anti-inflammatory activity (up to 63.4%, 62.0%, 64.1% and 62.5% edema inhibition, respectively), as compared to the standard drug diclofenac (67.0%). Pathological investigation has shown that the analogues of phenoxy ethanoic acid compounds have higher anti hyperlipidmeic effect and caused appropriate modulation in HDL levels and some compounds showed a good potential for obesity-associated hyperlipidemia (Khanum *et al.*, 2010).The phenoxy acetic acid analogues show very good antitumor activity on Ehrlich asites tumor cells (Prabhakar *et al.*, 2006) and also show the anti ulcerogenic activity, cyclooxygenase activity, anticonvulsant activity (Sudha *et al.*, 2003) and anti microbial activities (Ma *et al.*, 2011). We were interested in obtaining these type of compounds to evaluate their biological activity; for this purpose, the title compound,[4-(2-chlorobenzoyl)-2,6-dimethylphenoxy].ethanoic acid (I) was synthesized.

The asymmetric unit of (I) comprises two crystallographically independent molecules, A and B,respectively (Fig. 1). The geometry of both independent molecules indicates a high degree of similarity in terms of bond distances and angles. The average aromatic bond length in the phenyl ring is 1.381 (3) Å and 1.380 (3) Å for A, B, respectively. Remaining bond distances are within normal ranges (Allen *et al.*, 1987). The dihedral angle between the phenyl rings is 63.41 (8)° for molecule A and 61.41 (9)° for molecule B. Adjacent molecules of different type (A,B) are interconnected in pairs, through π-π interactions between their central phenyl rings (C1-C6) [ centroid separation = 3.801 (2)Å, interplanar spacing = 3.605Å, centroid shift = 1.204Å]. Finally, C—H···O hydrogen bonds (Table 1) link these dimeric entities into bilayered structures parallel to (100).

### Experimental

[4-(2-chlorobenzoyl)-2,6-dimethylphenoxy]ethanoic acid was obtained by refluxing a mixture of(2-Chloro-phenyl)-(4-hydroxy-3,5-dimethyl-phenyl)-methanone (1 g, 0.0038 mol) and ethyl chloroacetate (1.41 g, 0.011 mol) in dry acetone (50 ml) and anhydrous potassium carbonate (1.59 g, 0.0114 mol) for 14 hrs. The reaction mixture was cooled, and the solvent was removed by distillation. The residual mass was triturated with cold water to remove potassium carbonate, and extracted with ether (3 *X* 50 ml). The ether layer was washed with a 10% sodium hydroxide solution (3 *X* 50 ml), followed by water (3 *X* 30 ml), and then dried over anhydrous sodium sulfate and evaporated to dryness to obtain the product. Which on recrystallization with ethanol, gave [4-(2-chlorobenzoyl)-2,6-dimethylphenoxy]ethanoic acid with 85% yield. *M*.p.70–72°C; IR (Nujol): 1745 (ester, C O), 1665 cm-1 (C O); 1H NMR (CDCl3): δ 1.2 (t, J = 7 Hz, 3H, CH3 of ester), 2.3 (s, 6H, 2Ar-CH3), 4.25 (q, J = 6 Hz, 2H, CH2 of ester), 4.45 (s, 2H, OCH2), 7.2–7.8 (bm, 6H, Ar—H). Anal. Cal. for C19H19ClO4 (346.10): C, 65.80; H, 5.52; Found: C, 65.85; H, 5.58%.

### Refinement

All H atoms were positioned geometrically and were treated as riding on their parent C atoms, with C—H distances of 0.93–0.97 Å; and with *U*_iso_(H) = 1.2*U*_eq_(C), except for the methyl groups where *U*_iso_(H) = 1.5*U*_eq_(C).

### Crystal data

**Table:**

C_19_H_19_ClO_4_	*F*(000) = 1456
*M**_r_* = 346.79	*D*_x_ = 1.292 Mg m^−^^3^
Monoclinic, *P*2_1_/*c*	Mo *K*α radiation, λ = 0.71073 Å
Hall symbol: -P 2ybc	Cell parameters from 5654 reflections
*a* = 16.4082 (8) Å	θ = 3.5–28.9°
*b* = 14.7290 (6) Å	µ = 0.23 mm^−^^1^
*c* = 15.4470 (8) Å	*T* = 293 K
β = 107.268 (5)°	Block-shaped, white
*V* = 3564.9 (3) Å^3^	0.30 × 0.20 × 0.20 mm
*Z* = 8	

### Data collection

**Table:**

Oxford Diffraction Xcalibur Sapphire3 diffractometer	6997 independent reflections
Radiation source: fine-focus sealed tube	3576 reflections with *I* > 2σ(*I*)
Graphite monochromator	*R*_int_ = 0.039
Detector resolution: 0 pixels mm^-1^	θ_max_ = 26.0°, θ_min_ = 3.5°
ω scans	*h* = −20→18
Absorption correction: multi-scan (*CrysAlis PRO*; Oxford Diffraction, 2010)	*k* = −18→17
*T*_min_ = 0.912, *T*_max_ = 1.000	*l* = −12→19
17025 measured reflections	

### Refinement

**Table:**

Refinement on *F*^2^	Primary atom site location: structure-invariant direct methods
Least-squares matrix: full	Secondary atom site location: difference Fourier map
*R*[*F*^2^ > 2σ(*F*^2^)] = 0.064	Hydrogen site location: inferred from neighbouring sites
*wR*(*F*^2^) = 0.191	H-atom parameters constrained
*S* = 1.02	*w* = 1/[σ^2^(*F*_o_^2^) + (0.0686*P*)^2^ + 0.8701*P*] where *P* = (*F*_o_^2^ + 2*F*_c_^2^)/3
6997 reflections	(Δ/σ)_max_ = 0.001
439 parameters	Δρ_max_ = 0.46 e Å^−^^3^
0 restraints	Δρ_min_ = −0.36 e Å^−^^3^

### Special details

**Table:**

Experimental. *CrysAlis PRO*, Oxford Diffraction Ltd., Version 1.171.34.40 (release 27–08-2010 CrysAlis171. NET) (compiled Aug 27 2010,11:50:40) Empirical absorption correction using spherical harmonics, implemented in SCALE3 ABSPACK scaling algorithm.
Geometry. All e.s.d.'s (except the e.s.d. in the dihedral angle between two l.s. planes) are estimated using the full covariance matrix. The cell e.s.d.'s are taken into account individually in the estimation of e.s.d.'s in distances, angles and torsion angles; correlations between e.s.d.'s in cell parameters are only used when they are defined by crystal symmetry. An approximate (isotropic) treatment of cell e.s.d.'s is used for estimating e.s.d.'s involving l.s. planes.
Refinement. Refinement of *F*^2^ against ALL reflections. The weighted *R*-factor *wR* and goodness of fit *S* are based on *F*^2^, conventional *R*-factors *R* are based on *F*, with *F* set to zero for negative *F*^2^. The threshold expression of *F*^2^ > σ(*F*^2^) is used only for calculating *R*-factors(gt) *etc*. and is not relevant to the choice of reflections for refinement. *R*-factors based on *F*^2^ are statistically about twice as large as those based on *F*, and *R*- factors based on ALL data will be even larger.

### Fractional atomic coordinates and isotropic or equivalent isotropic displacement parameters (Å^2^)

**Table:**

	*x*	*y*	*z*	*U*_iso_*/*U*_eq_	
Cl1A	0.46159 (6)	0.21534 (6)	0.22421 (7)	0.0684 (3)	
Cl1B	−0.03423 (6)	0.22771 (7)	−0.02662 (7)	0.0685 (3)	
O16A	0.28452 (14)	0.50209 (13)	−0.06982 (16)	0.0558 (6)	
O16B	−0.20126 (16)	0.51127 (14)	−0.31401 (17)	0.0647 (7)	
O9A	0.21270 (14)	0.22608 (16)	0.21320 (16)	0.0665 (7)	
C4A	0.27950 (18)	0.33354 (18)	0.1437 (2)	0.0394 (7)	
C9A	0.27483 (19)	0.2738 (2)	0.2186 (2)	0.0440 (8)	
C10A	0.34554 (19)	0.27262 (18)	0.3070 (2)	0.0401 (7)	
O9B	−0.28615 (15)	0.23574 (18)	−0.03864 (17)	0.0755 (8)	
C15B	−0.0681 (2)	0.25322 (19)	0.0673 (2)	0.0437 (8)	
C10B	−0.15203 (19)	0.27675 (19)	0.0581 (2)	0.0411 (7)	
C5A	0.21687 (18)	0.32661 (19)	0.0610 (2)	0.0450 (8)	
H5A	0.1738	0.2837	0.0541	0.054*	
O19A	0.36454 (17)	0.50616 (15)	−0.25734 (17)	0.0687 (7)	
C15A	0.4298 (2)	0.24726 (19)	0.3174 (2)	0.0456 (8)	
C9B	−0.22215 (19)	0.2815 (2)	−0.0310 (2)	0.0472 (8)	
C1B	−0.2065 (2)	0.4546 (2)	−0.2440 (2)	0.0497 (8)	
C1A	0.28278 (19)	0.44450 (19)	0.0007 (2)	0.0441 (8)	
C4B	−0.21539 (18)	0.34356 (19)	−0.1037 (2)	0.0419 (7)	
C5B	−0.27603 (19)	0.3376 (2)	−0.1884 (2)	0.0492 (8)	
H5B	−0.3195	0.2950	−0.1971	0.059*	
C2B	−0.1455 (2)	0.46487 (19)	−0.1604 (2)	0.0491 (8)	
C2A	0.34656 (19)	0.45378 (19)	0.0823 (2)	0.0457 (8)	
C6A	0.21651 (18)	0.3816 (2)	−0.0115 (2)	0.0458 (8)	
C3A	0.34300 (19)	0.39839 (19)	0.1539 (2)	0.0457 (8)	
H3A	0.3841	0.4049	0.2098	0.055*	
C6B	−0.2738 (2)	0.3923 (2)	−0.2594 (2)	0.0525 (9)	
C3B	−0.15142 (18)	0.40863 (19)	−0.0898 (2)	0.0462 (8)	
H3B	−0.1120	0.4148	−0.0327	0.055*	
O19B	−0.1394 (2)	0.49360 (17)	−0.5120 (2)	0.0873 (9)	
C14A	0.4899 (2)	0.2435 (2)	0.4019 (3)	0.0575 (10)	
H14A	0.5462	0.2283	0.4076	0.069*	
O18A	0.3528 (2)	0.61754 (18)	−0.1649 (2)	0.1018 (11)	
C11A	0.3232 (2)	0.2926 (2)	0.3852 (2)	0.0532 (9)	
H11A	0.2675	0.3101	0.3803	0.064*	
C11B	−0.1756 (2)	0.2909 (2)	0.1363 (2)	0.0550 (9)	
H11B	−0.2318	0.3056	0.1317	0.066*	
C14B	−0.0086 (2)	0.2459 (2)	0.1512 (3)	0.0548 (9)	
H14B	0.0475	0.2302	0.1563	0.066*	
C8A	0.1461 (2)	0.3761 (3)	−0.1005 (2)	0.0692 (11)	
H8A1	0.1649	0.3402	−0.1427	0.104*	
H8A2	0.1320	0.4362	−0.1245	0.104*	
H8A3	0.0965	0.3486	−0.0906	0.104*	
C12B	−0.1167 (3)	0.2836 (2)	0.2201 (3)	0.0658 (10)	
H12B	−0.1332	0.2932	0.2720	0.079*	
C18B	−0.1566 (3)	0.5337 (3)	−0.4445 (3)	0.0716 (12)	
C18A	0.3471 (2)	0.5397 (2)	−0.1853 (3)	0.0555 (9)	
O18B	−0.1572 (3)	0.6142 (2)	−0.4361 (3)	0.1385 (16)	
C12A	0.3822 (3)	0.2869 (2)	0.4696 (3)	0.0679 (11)	
H12A	0.3662	0.2994	0.5213	0.081*	
C13A	0.4648 (3)	0.2625 (3)	0.4770 (3)	0.0699 (11)	
H13A	0.5045	0.2589	0.5341	0.084*	
C17A	0.3229 (2)	0.4645 (2)	−0.1325 (2)	0.0577 (9)	
H17A	0.2832	0.4236	−0.1733	0.069*	
H17B	0.3732	0.4302	−0.1004	0.069*	
C17B	−0.1633 (3)	0.4689 (3)	−0.3750 (3)	0.0862 (14)	
H17C	−0.1977	0.4174	−0.4036	0.103*	
H17D	−0.1070	0.4468	−0.3420	0.103*	
C13B	−0.0333 (2)	0.2619 (2)	0.2274 (3)	0.0662 (11)	
H13B	0.0067	0.2581	0.2844	0.079*	
C7A	0.4158 (2)	0.5230 (2)	0.0931 (3)	0.0693 (11)	
H7A1	0.4576	0.5006	0.0662	0.104*	
H7A2	0.4425	0.5343	0.1564	0.104*	
H7A3	0.3917	0.5784	0.0637	0.104*	
C7B	−0.0761 (2)	0.5349 (2)	−0.1455 (3)	0.0763 (12)	
H7B1	−0.0352	0.5161	−0.1753	0.115*	
H7B2	−0.0482	0.5414	−0.0817	0.115*	
H7B3	−0.1005	0.5920	−0.1700	0.115*	
C8B	−0.3420 (2)	0.3872 (3)	−0.3487 (3)	0.0811 (12)	
H8B1	−0.3215	0.3529	−0.3908	0.122*	
H8B2	−0.3567	0.4475	−0.3718	0.122*	
H8B3	−0.3916	0.3580	−0.3406	0.122*	
C20A	0.3913 (3)	0.5703 (3)	−0.3153 (3)	0.0890 (14)	
H20A	0.3461	0.6137	−0.3406	0.107*	
H20B	0.4413	0.6034	−0.2801	0.107*	
C21A	0.4110 (4)	0.5209 (3)	−0.3876 (3)	0.130 (2)	
H21A	0.4581	0.4806	−0.3622	0.195*	
H21B	0.4260	0.5629	−0.4278	0.195*	
H21C	0.3621	0.4862	−0.4205	0.195*	
C21B	−0.1070 (4)	0.4990 (3)	−0.6498 (4)	0.138 (2)	
H21D	−0.0524	0.4718	−0.6219	0.208*	
H21E	−0.1033	0.5380	−0.6983	0.208*	
H21F	−0.1484	0.4523	−0.6735	0.208*	
C20B	−0.1313 (4)	0.5491 (3)	−0.5865 (3)	0.1206 (19)	
H20C	−0.1855	0.5783	−0.6155	0.145*	
H20D	−0.0894	0.5963	−0.5629	0.145*	

### Atomic displacement parameters (Å^2^)

**Table:**

	*U*^11^	*U*^22^	*U*^33^	*U*^12^	*U*^13^	*U*^23^
Cl1A	0.0703 (6)	0.0780 (6)	0.0640 (7)	0.0168 (5)	0.0309 (5)	−0.0039 (5)
Cl1B	0.0679 (6)	0.0860 (7)	0.0604 (7)	0.0089 (5)	0.0325 (5)	−0.0015 (5)
O16A	0.0690 (15)	0.0503 (13)	0.0549 (16)	0.0154 (11)	0.0288 (12)	0.0158 (11)
O16B	0.0882 (17)	0.0547 (14)	0.0625 (18)	0.0231 (12)	0.0395 (14)	0.0205 (12)
O9A	0.0536 (14)	0.0836 (17)	0.0555 (18)	−0.0271 (13)	0.0059 (12)	0.0114 (13)
C4A	0.0405 (16)	0.0386 (16)	0.0380 (19)	−0.0016 (13)	0.0098 (14)	−0.0001 (13)
C9A	0.0446 (18)	0.0472 (18)	0.040 (2)	−0.0020 (15)	0.0119 (15)	0.0025 (14)
C10A	0.0467 (17)	0.0334 (15)	0.039 (2)	−0.0034 (13)	0.0113 (15)	0.0009 (13)
O9B	0.0594 (16)	0.0992 (19)	0.0598 (19)	−0.0345 (14)	0.0054 (14)	0.0160 (14)
C15B	0.0485 (18)	0.0441 (18)	0.040 (2)	−0.0042 (14)	0.0154 (16)	0.0005 (13)
C10B	0.0467 (18)	0.0394 (17)	0.036 (2)	−0.0062 (14)	0.0111 (15)	0.0020 (13)
C5A	0.0452 (17)	0.0465 (18)	0.044 (2)	−0.0042 (14)	0.0132 (15)	−0.0002 (15)
O19A	0.0955 (19)	0.0601 (15)	0.0593 (18)	−0.0042 (13)	0.0369 (15)	0.0092 (12)
C15A	0.0504 (19)	0.0429 (18)	0.043 (2)	−0.0025 (14)	0.0138 (17)	−0.0053 (14)
C9B	0.0463 (18)	0.0508 (19)	0.044 (2)	−0.0057 (15)	0.0130 (16)	0.0009 (15)
C1B	0.057 (2)	0.0479 (19)	0.049 (2)	0.0156 (16)	0.0243 (17)	0.0142 (16)
C1A	0.0502 (18)	0.0387 (17)	0.047 (2)	0.0072 (14)	0.0201 (16)	0.0057 (15)
C4B	0.0386 (16)	0.0462 (18)	0.040 (2)	−0.0020 (14)	0.0102 (14)	0.0013 (14)
C5B	0.0445 (17)	0.059 (2)	0.044 (2)	−0.0072 (15)	0.0115 (16)	0.0003 (16)
C2B	0.0501 (18)	0.0399 (17)	0.060 (2)	0.0015 (15)	0.0198 (17)	0.0027 (16)
C2A	0.0481 (18)	0.0373 (17)	0.052 (2)	−0.0021 (14)	0.0157 (16)	0.0009 (15)
C6A	0.0417 (17)	0.0555 (19)	0.040 (2)	0.0054 (15)	0.0113 (15)	0.0025 (15)
C3A	0.0443 (17)	0.0463 (18)	0.044 (2)	−0.0013 (15)	0.0088 (15)	−0.0032 (14)
C6B	0.052 (2)	0.064 (2)	0.040 (2)	0.0098 (17)	0.0119 (16)	0.0027 (16)
C3B	0.0420 (17)	0.0493 (19)	0.043 (2)	−0.0022 (14)	0.0053 (15)	0.0021 (15)
O19B	0.136 (3)	0.0667 (17)	0.076 (2)	−0.0011 (16)	0.058 (2)	0.0106 (15)
C14A	0.050 (2)	0.057 (2)	0.058 (3)	0.0012 (16)	0.004 (2)	0.0032 (17)
O18A	0.148 (3)	0.0523 (16)	0.142 (3)	−0.0045 (17)	0.101 (2)	0.0005 (17)
C11A	0.058 (2)	0.058 (2)	0.046 (2)	−0.0006 (16)	0.0203 (18)	−0.0063 (16)
C11B	0.064 (2)	0.058 (2)	0.047 (2)	0.0021 (17)	0.0223 (18)	0.0008 (17)
C14B	0.051 (2)	0.057 (2)	0.051 (3)	0.0015 (16)	0.0078 (19)	0.0062 (16)
C8A	0.058 (2)	0.096 (3)	0.047 (2)	−0.005 (2)	0.0058 (18)	0.007 (2)
C12B	0.084 (3)	0.079 (3)	0.038 (2)	0.002 (2)	0.024 (2)	−0.0026 (19)
C18B	0.093 (3)	0.053 (2)	0.086 (3)	0.018 (2)	0.054 (3)	0.019 (2)
C18A	0.055 (2)	0.047 (2)	0.070 (3)	0.0069 (16)	0.0275 (19)	0.0076 (18)
O18B	0.237 (4)	0.072 (2)	0.160 (4)	0.033 (2)	0.141 (3)	0.031 (2)
C12A	0.082 (3)	0.080 (3)	0.042 (2)	−0.008 (2)	0.020 (2)	−0.0121 (19)
C13A	0.070 (3)	0.080 (3)	0.046 (3)	−0.008 (2)	−0.004 (2)	0.0036 (19)
C17A	0.073 (2)	0.052 (2)	0.053 (2)	0.0069 (17)	0.0262 (19)	0.0067 (17)
C17B	0.128 (4)	0.070 (3)	0.086 (3)	0.024 (2)	0.070 (3)	0.023 (2)
C13B	0.070 (3)	0.074 (2)	0.041 (3)	−0.001 (2)	−0.003 (2)	0.0093 (18)
C7A	0.068 (2)	0.064 (2)	0.074 (3)	−0.0168 (19)	0.018 (2)	0.007 (2)
C7B	0.072 (2)	0.064 (2)	0.094 (3)	−0.011 (2)	0.026 (2)	0.015 (2)
C8B	0.075 (3)	0.112 (3)	0.048 (3)	0.005 (2)	0.006 (2)	0.010 (2)
C20A	0.124 (4)	0.077 (3)	0.082 (3)	0.006 (3)	0.054 (3)	0.029 (2)
C21A	0.228 (7)	0.103 (4)	0.093 (4)	−0.009 (4)	0.097 (4)	0.014 (3)
C21B	0.245 (7)	0.112 (4)	0.078 (4)	0.033 (4)	0.079 (5)	0.019 (3)
C20B	0.192 (6)	0.105 (4)	0.083 (4)	−0.006 (4)	0.069 (4)	0.032 (3)

### Geometric parameters (Å, º)

**Table:**

Cl1A—C15A	1.736 (3)	O18A—C18A	1.186 (4)
Cl1B—C15B	1.742 (3)	C11A—C12A	1.377 (4)
O16A—C1A	1.387 (3)	C11A—H11A	0.9300
O16A—C17A	1.414 (4)	C11B—C12B	1.370 (5)
O16B—C1B	1.389 (4)	C11B—H11B	0.9300
O16B—C17B	1.419 (4)	C14B—C13B	1.374 (5)
O9A—C9A	1.220 (3)	C14B—H14B	0.9300
C4A—C5A	1.386 (4)	C8A—H8A1	0.9600
C4A—C3A	1.388 (4)	C8A—H8A2	0.9600
C4A—C9A	1.474 (4)	C8A—H8A3	0.9600
C9A—C10A	1.507 (4)	C12B—C13B	1.378 (5)
C10A—C15A	1.394 (4)	C12B—H12B	0.9300
C10A—C11A	1.394 (4)	C18B—O18B	1.193 (4)
O9B—C9B	1.224 (3)	C18B—C17B	1.464 (5)
C15B—C14B	1.376 (4)	C18A—C17A	1.498 (4)
C15B—C10B	1.385 (4)	C12A—C13A	1.374 (5)
C10B—C11B	1.388 (4)	C12A—H12A	0.9300
C10B—C9B	1.511 (4)	C13A—H13A	0.9300
C5A—C6A	1.381 (4)	C17A—H17A	0.9700
C5A—H5A	0.9300	C17A—H17B	0.9700
O19A—C18A	1.323 (4)	C17B—H17C	0.9700
O19A—C20A	1.457 (4)	C17B—H17D	0.9700
C15A—C14A	1.384 (4)	C13B—H13B	0.9300
C9B—C4B	1.477 (4)	C7A—H7A1	0.9600
C1B—C2B	1.388 (4)	C7A—H7A2	0.9600
C1B—C6B	1.400 (4)	C7A—H7A3	0.9600
C1A—C2A	1.385 (4)	C7B—H7B1	0.9600
C1A—C6A	1.398 (4)	C7B—H7B2	0.9600
C4B—C3B	1.390 (4)	C7B—H7B3	0.9600
C4B—C5B	1.392 (4)	C8B—H8B1	0.9600
C5B—C6B	1.370 (4)	C8B—H8B2	0.9600
C5B—H5B	0.9300	C8B—H8B3	0.9600
C2B—C3B	1.396 (4)	C20A—C21A	1.448 (6)
C2B—C7B	1.502 (4)	C20A—H20A	0.9700
C2A—C3A	1.389 (4)	C20A—H20B	0.9700
C2A—C7A	1.499 (4)	C21A—H21A	0.9600
C6A—C8A	1.512 (4)	C21A—H21B	0.9600
C3A—H3A	0.9300	C21A—H21C	0.9600
C6B—C8B	1.498 (4)	C21B—C20B	1.376 (6)
C3B—H3B	0.9300	C21B—H21D	0.9600
O19B—C18B	1.300 (4)	C21B—H21E	0.9600
O19B—C20B	1.450 (5)	C21B—H21F	0.9600
C14A—C13A	1.369 (5)	C20B—H20C	0.9700
C14A—H14A	0.9300	C20B—H20D	0.9700
			
C1A—O16A—C17A	114.5 (2)	H8A1—C8A—H8A3	109.5
C1B—O16B—C17B	113.2 (2)	H8A2—C8A—H8A3	109.5
C5A—C4A—C3A	118.8 (3)	C11B—C12B—C13B	120.1 (4)
C5A—C4A—C9A	118.9 (3)	C11B—C12B—H12B	120.0
C3A—C4A—C9A	122.3 (3)	C13B—C12B—H12B	120.0
O9A—C9A—C4A	121.8 (3)	O18B—C18B—O19B	123.3 (4)
O9A—C9A—C10A	117.3 (3)	O18B—C18B—C17B	124.3 (4)
C4A—C9A—C10A	120.9 (3)	O19B—C18B—C17B	111.9 (3)
C15A—C10A—C11A	117.6 (3)	O18A—C18A—O19A	124.4 (3)
C15A—C10A—C9A	125.3 (3)	O18A—C18A—C17A	125.6 (4)
C11A—C10A—C9A	116.9 (3)	O19A—C18A—C17A	109.9 (3)
C14B—C15B—C10B	121.4 (3)	C13A—C12A—C11A	119.6 (4)
C14B—C15B—Cl1B	117.1 (3)	C13A—C12A—H12A	120.2
C10B—C15B—Cl1B	121.5 (3)	C11A—C12A—H12A	120.2
C15B—C10B—C11B	118.2 (3)	C14A—C13A—C12A	121.4 (4)
C15B—C10B—C9B	124.7 (3)	C14A—C13A—H13A	119.3
C11B—C10B—C9B	116.9 (3)	C12A—C13A—H13A	119.3
C6A—C5A—C4A	121.9 (3)	O16A—C17A—C18A	109.1 (3)
C6A—C5A—H5A	119.1	O16A—C17A—H17A	109.9
C4A—C5A—H5A	119.1	C18A—C17A—H17A	109.9
C18A—O19A—C20A	117.0 (3)	O16A—C17A—H17B	109.9
C14A—C15A—C10A	121.6 (3)	C18A—C17A—H17B	109.9
C14A—C15A—Cl1A	117.8 (3)	H17A—C17A—H17B	108.3
C10A—C15A—Cl1A	120.6 (3)	O16B—C17B—C18B	110.0 (3)
O9B—C9B—C4B	121.6 (3)	O16B—C17B—H17C	109.7
O9B—C9B—C10B	117.5 (3)	C18B—C17B—H17C	109.7
C4B—C9B—C10B	120.8 (3)	O16B—C17B—H17D	109.7
C2B—C1B—O16B	117.6 (3)	C18B—C17B—H17D	109.7
C2B—C1B—C6B	123.0 (3)	H17C—C17B—H17D	108.2
O16B—C1B—C6B	119.3 (3)	C14B—C13B—C12B	120.5 (3)
C2A—C1A—O16A	117.9 (3)	C14B—C13B—H13B	119.8
C2A—C1A—C6A	122.4 (3)	C12B—C13B—H13B	119.8
O16A—C1A—C6A	119.7 (3)	C2A—C7A—H7A1	109.5
C3B—C4B—C5B	119.0 (3)	C2A—C7A—H7A2	109.5
C3B—C4B—C9B	122.2 (3)	H7A1—C7A—H7A2	109.5
C5B—C4B—C9B	118.8 (3)	C2A—C7A—H7A3	109.5
C6B—C5B—C4B	122.3 (3)	H7A1—C7A—H7A3	109.5
C6B—C5B—H5B	118.9	H7A2—C7A—H7A3	109.5
C4B—C5B—H5B	118.9	C2B—C7B—H7B1	109.5
C1B—C2B—C3B	117.6 (3)	C2B—C7B—H7B2	109.5
C1B—C2B—C7B	121.5 (3)	H7B1—C7B—H7B2	109.5
C3B—C2B—C7B	120.9 (3)	C2B—C7B—H7B3	109.5
C1A—C2A—C3A	117.9 (3)	H7B1—C7B—H7B3	109.5
C1A—C2A—C7A	120.8 (3)	H7B2—C7B—H7B3	109.5
C3A—C2A—C7A	121.3 (3)	C6B—C8B—H8B1	109.5
C5A—C6A—C1A	117.6 (3)	C6B—C8B—H8B2	109.5
C5A—C6A—C8A	121.6 (3)	H8B1—C8B—H8B2	109.5
C1A—C6A—C8A	120.8 (3)	C6B—C8B—H8B3	109.5
C4A—C3A—C2A	121.4 (3)	H8B1—C8B—H8B3	109.5
C4A—C3A—H3A	119.3	H8B2—C8B—H8B3	109.5
C2A—C3A—H3A	119.3	C21A—C20A—O19A	109.0 (3)
C5B—C6B—C1B	117.2 (3)	C21A—C20A—H20A	109.9
C5B—C6B—C8B	121.2 (3)	O19A—C20A—H20A	109.9
C1B—C6B—C8B	121.7 (3)	C21A—C20A—H20B	109.9
C4B—C3B—C2B	120.9 (3)	O19A—C20A—H20B	109.9
C4B—C3B—H3B	119.5	H20A—C20A—H20B	108.3
C2B—C3B—H3B	119.5	C20A—C21A—H21A	109.5
C18B—O19B—C20B	118.3 (3)	C20A—C21A—H21B	109.5
C13A—C14A—C15A	118.8 (3)	H21A—C21A—H21B	109.5
C13A—C14A—H14A	120.6	C20A—C21A—H21C	109.5
C15A—C14A—H14A	120.6	H21A—C21A—H21C	109.5
C12A—C11A—C10A	121.0 (3)	H21B—C21A—H21C	109.5
C12A—C11A—H11A	119.5	C20B—C21B—H21D	109.5
C10A—C11A—H11A	119.5	C20B—C21B—H21E	109.5
C12B—C11B—C10B	120.6 (3)	H21D—C21B—H21E	109.5
C12B—C11B—H11B	119.7	C20B—C21B—H21F	109.5
C10B—C11B—H11B	119.7	H21D—C21B—H21F	109.5
C13B—C14B—C15B	119.1 (3)	H21E—C21B—H21F	109.5
C13B—C14B—H14B	120.4	C21B—C20B—O19B	112.1 (4)
C15B—C14B—H14B	120.4	C21B—C20B—H20C	109.2
C6A—C8A—H8A1	109.5	O19B—C20B—H20C	109.2
C6A—C8A—H8A2	109.5	C21B—C20B—H20D	109.2
H8A1—C8A—H8A2	109.5	O19B—C20B—H20D	109.2
C6A—C8A—H8A3	109.5	H20C—C20B—H20D	107.9
			
C5A—C4A—C9A—O9A	−8.5 (5)	O16A—C1A—C6A—C5A	−179.1 (3)
C3A—C4A—C9A—O9A	169.5 (3)	C2A—C1A—C6A—C8A	177.5 (3)
C5A—C4A—C9A—C10A	174.3 (3)	O16A—C1A—C6A—C8A	−0.6 (5)
C3A—C4A—C9A—C10A	−7.6 (4)	C5A—C4A—C3A—C2A	−2.3 (5)
O9A—C9A—C10A—C15A	120.5 (4)	C9A—C4A—C3A—C2A	179.7 (3)
C4A—C9A—C10A—C15A	−62.2 (4)	C1A—C2A—C3A—C4A	2.2 (5)
O9A—C9A—C10A—C11A	−54.9 (4)	C7A—C2A—C3A—C4A	−179.3 (3)
C4A—C9A—C10A—C11A	122.4 (3)	C4B—C5B—C6B—C1B	1.0 (5)
C14B—C15B—C10B—C11B	−1.4 (4)	C4B—C5B—C6B—C8B	−177.3 (3)
Cl1B—C15B—C10B—C11B	176.3 (2)	C2B—C1B—C6B—C5B	−2.5 (5)
C14B—C15B—C10B—C9B	−176.6 (3)	O16B—C1B—C6B—C5B	179.7 (3)
Cl1B—C15B—C10B—C9B	1.0 (4)	C2B—C1B—C6B—C8B	175.8 (3)
C3A—C4A—C5A—C6A	0.7 (5)	O16B—C1B—C6B—C8B	−2.0 (5)
C9A—C4A—C5A—C6A	178.8 (3)	C5B—C4B—C3B—C2B	−2.6 (5)
C11A—C10A—C15A—C14A	−1.2 (4)	C9B—C4B—C3B—C2B	178.5 (3)
C9A—C10A—C15A—C14A	−176.5 (3)	C1B—C2B—C3B—C4B	1.2 (5)
C11A—C10A—C15A—Cl1A	176.5 (2)	C7B—C2B—C3B—C4B	−179.7 (3)
C9A—C10A—C15A—Cl1A	1.2 (4)	C10A—C15A—C14A—C13A	2.0 (5)
C15B—C10B—C9B—O9B	124.0 (4)	Cl1A—C15A—C14A—C13A	−175.7 (3)
C11B—C10B—C9B—O9B	−51.3 (4)	C15A—C10A—C11A—C12A	−0.4 (4)
C15B—C10B—C9B—C4B	−59.7 (4)	C9A—C10A—C11A—C12A	175.3 (3)
C11B—C10B—C9B—C4B	125.0 (3)	C15B—C10B—C11B—C12B	1.3 (4)
C17B—O16B—C1B—C2B	100.3 (4)	C9B—C10B—C11B—C12B	176.9 (3)
C17B—O16B—C1B—C6B	−81.8 (4)	C10B—C15B—C14B—C13B	0.2 (5)
C17A—O16A—C1A—C2A	97.0 (3)	Cl1B—C15B—C14B—C13B	−177.5 (3)
C17A—O16A—C1A—C6A	−84.8 (4)	C10B—C11B—C12B—C13B	0.0 (5)
O9B—C9B—C4B—C3B	167.1 (3)	C20B—O19B—C18B—O18B	−8.1 (7)
C10B—C9B—C4B—C3B	−9.1 (5)	C20B—O19B—C18B—C17B	179.7 (4)
O9B—C9B—C4B—C5B	−11.8 (5)	C20A—O19A—C18A—O18A	−0.9 (6)
C10B—C9B—C4B—C5B	172.0 (3)	C20A—O19A—C18A—C17A	−178.6 (3)
C3B—C4B—C5B—C6B	1.5 (5)	C10A—C11A—C12A—C13A	1.1 (5)
C9B—C4B—C5B—C6B	−179.6 (3)	C15A—C14A—C13A—C12A	−1.3 (5)
O16B—C1B—C2B—C3B	179.2 (3)	C11A—C12A—C13A—C14A	−0.2 (6)
C6B—C1B—C2B—C3B	1.4 (5)	C1A—O16A—C17A—C18A	−161.4 (3)
O16B—C1B—C2B—C7B	0.2 (5)	O18A—C18A—C17A—O16A	16.7 (5)
C6B—C1B—C2B—C7B	−177.6 (3)	O19A—C18A—C17A—O16A	−165.6 (3)
O16A—C1A—C2A—C3A	177.7 (3)	C1B—O16B—C17B—C18B	−177.6 (3)
C6A—C1A—C2A—C3A	−0.5 (5)	O18B—C18B—C17B—O16B	21.5 (7)
O16A—C1A—C2A—C7A	−0.9 (4)	O19B—C18B—C17B—O16B	−166.3 (4)
C6A—C1A—C2A—C7A	−179.1 (3)	C15B—C14B—C13B—C12B	1.1 (5)
C4A—C5A—C6A—C1A	0.9 (5)	C11B—C12B—C13B—C14B	−1.2 (5)
C4A—C5A—C6A—C8A	−177.6 (3)	C18A—O19A—C20A—C21A	177.5 (4)
C2A—C1A—C6A—C5A	−1.0 (5)	C18B—O19B—C20B—C21B	175.6 (5)

### Hydrogen-bond geometry (Å, º)

**Table:**

*D*—H···*A*	*D*—H	H···*A*	*D*···*A*	*D*—H···*A*
C11*A*—H11*A*···O18*B*^i^	0.93	2.49	3.346 (6)	153
C11*B*—H11*B*···O18*A*^i^	0.93	2.47	3.351 (5)	159
C14*B*—H14*B*···O9*A*	0.93	2.59	3.482 (4)	161
C20*A*—H20*A*···O9*B*^ii^	0.97	2.57	3.420 (5)	147

Symmetry codes: (i) −*x*, −*y*+1, −*z*; (ii) −*x*, *y*+1/2, −*z*−1/2.
